# Diagnostic tomodensitométrique d’une hernie de Spiegel étranglée: à propos d’une observation

**DOI:** 10.11604/pamj.2016.25.222.10298

**Published:** 2016-12-07

**Authors:** Geraud Akpo, Hamidou Deme, Nfally Badji, Fallou Niang, Mohamadou Toure, Ibrahima Niang, Malick Diouf, El Hadj Niang

**Affiliations:** 1 Service de Radiologie et d’Imagerie Médicale de l’Hôpital Aristide Le Dantec, Dakar, Sénégal

**Keywords:** Hernie Spiegel, tomodensitométrie, étranglement, Spiegel hernia, CT scan, strangulation

## Abstract

Nous rapportons un cas de hernie de Spiegel compliquée d'occlusion chez une femme de 86 ans dont le diagnostic a été posé à la tomodensitométrie Elle avait consulté aux urgences chirurgicales pour des douleurs de la fosse iliaque droite d'apparitions brutales associées à des vomissements. L'examen physique a retrouvé une patiente fébrile (38,2°), une masse localisée à la fosse iliaque droite ferme, sensible et mobile par rapport aux deux plans. La tomodensitométrie abdominale avait objectivé au niveau de la fosse iliaque droite, en avant de l'aponévrose du muscle oblique externe, un sac herniaire avec un collet mesuré à 13 mm. Il contenait de la graisse et une anse grêle en arceau présentant deux zones de transition donnant un aspect de double bec au niveau du collet. La paroi de l'anse incarcérée ne se rehaussait pas après injection de produit de contraste. Le diagnostic de hernie de spiegel étranglée avec signe d'ischémie artérielle de la paroi digestive a été retenu. Le traitement a été chirurgical avec des suites opératoires simples.

## Abstract (en)

We report a case of a 86-year old woman with Spiegel hernia complicated by occlusion whose diagnosis was based on CT scan. She was examined in the Emergency Surgery Department for brutal onset of pain in the right iliac fossa associated with vomiting. On physical examination the patient was febrile (38.2° C). It showed hard, sensitive and mobile mass located in the right iliac fossa, with respect to both planes. Abdominal CT scan showed a hernia sac with the neck measuring 13 mm in the right iliac fossa, in front of the aponeurosis of the external oblique muscle. It contained fat and a small bowel loop (curved arrow) with two zones of transition giving a double beak-like appearance at the level of the neck. CT scan showed a lack of enhancement of the wall of the loop after administration of contrast material. The diagnosis of strangulated spiegel hernia associated with sign of arterial ischemia of the digestive wall was retained. Surgery was perfomed with simple postoperative management.

## Introduction

La hernie de Spiegel est rare et représente 0,1% des hernies [1]. Elle est asymptomatique dans 90% des cas et son diagnostic positif est radiologique. La complication la plus fréquente est l'étranglement herniaire à l'origine d'une occlusion intestinale aiguë [2]. Nous rapportons un cas de hernie de Spiegel compliquée d'occlusion chez une femme de 86 ans dont le diagnostic a été posé à la tomodensitométrie.

## Patient et observation

**Figure 1:**
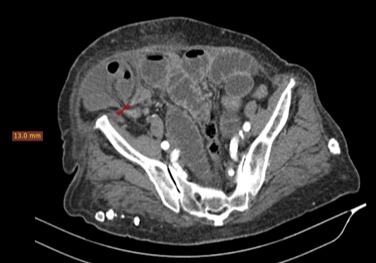
Coupe axiale TDM après injection de produit de contraste iodé montrant l’aspect en « double bec » de l’anse incarcérée au niveau du collet de la hernie

**Figure 2:**
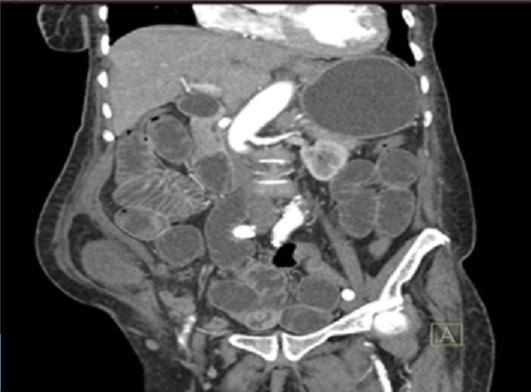
TDM abdominale après injection de produit de contraste iodé. Reconstructions coronales montrant une solution de continuité au niveau de la ligne de Spiegel. Présence d’un sac herniaire en situation interpariétale, contenant une anse grêle sans rehaussement de leur paroi

**Figure 3:**
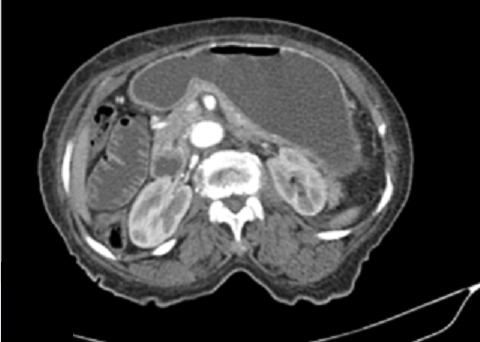
TDM abdominale après injection de produit de contraste iodé. Coupes axiales montrant une distension des anses grêles et de l’estomac consécutive à l’incarcération herniaire

**Figure 4:**
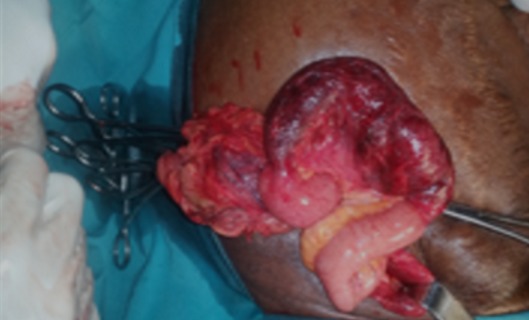
Image per opératoire confirmant la hernie de Spiegel et la nécrose du segment digestif incarcéré

Il s'agissait d'une femme de 86 ans, huit gestes et huit pares sans autre antécédent pathologique particulier. Elle avait consulté aux urgences chirurgicales pour des douleurs de la fosse iliaque droite d'apparition brutale, associées à des vomissements. L'examen physique a trouvé une patiente en bon état général, fébrile (38,2°), présentant un abdomen distendu et une masse localisée à la fosse iliaque droite. Elle était mobile par rapport aux deux plans, ferme et sensible. Le reste de l'examen était sans particularité. La tomodensitométrie abdominale avait objectivé au niveau de la fosse iliaque droite, en avant de l'aponévrose du muscle oblique externe, un sac herniaire de 84x56 mm de diamètre dans le plan axial pour une hauteur de 103 mm, avec un collet mesuré à 13 mm. Ce sac contenait de la graisse et une anse grêle en arceau présentant deux zones de transition donnant un aspect de double bec au niveau du collet (Figure 1). La paroi de cette anse incarcérée n'était pas rehaussée après injection de produit de contraste (Figure 2). On notait en amont une distension des anses grêles mesurant 33 mm et un aspect plat du colon en aval (Figure 3). Il n'y avait pas d'épanchement péritonéal ni de pneumopéritoine. Le diagnostic de hernie de spiegel étranglée avec signe d'ischémie artérielle de la paroi digestive a été retenu. L'exploration chirurgicale avait retrouvé un sac herniaire avec un collet étroit (3 cm), contenant de l'épiploon et une anse grêle nécrosée sur 5 cm. Une résection de l'épiploon et de l'anse incarcérée a été réalisée avec une anastomose termino-terminale, suivie d'une fermeture de l'orifice du sac et du défect musculaire (Figure 4). Les suites opératoires étaient simples.

## Discussion

Les hernies de Spiegel sont rares [1, 2]. Elles correspondent à la protrusion d'un sac péritonéal à travers un orifice anatomique acquis ou congénital de la ligne de Spiegel [1]. Elle survient généralement après l'âge de 40 ans [3]. Il existe des facteurs prédisposant telle qu'une hyperpression intra abdominale secondaire à l'obésité morbide, les grossesses multiples et la toux chronique [4]. Notre patiente était une multi gestes, pluri pares sans autre facteur de risque associé. La hernie se développe en situation interpariétale entre le muscle oblique externe en avant et le muscle oblique interne et transverse en arrière. Dans la plupart des cas, le sac herniaire contient de l'épiploon, du grêle, le cæcum, l'appendice ou le sigmoïde [2, 5, 6]. Dans ce cas présent, le sac herniaire contenait de l'épiploon et du grêle. Le cæcum et le sigmoïde étaient plats. Le collet de la hernie est en général étroit de 0,5 à 2 cm, et de ce fait, il est responsable d'incarcérations et d'étranglements avec syndrome occlusif [2, 5]. Ce qui a été observé chez notre patiente. En effet le collet de la hernie était mesuré à 13 mm et on notait une distension des anses intestinales avec une zone de transition au niveau du collet réalisant «un aspect en double bec ».

La tomodensitométrie abdominale, avec une grande sensibilité, reste l'examen clé du diagnostic, elle permet de voir la déhiscence musculaire et le contenu du sac herniaire [7]. En effet elle permet une bonne étude des différentes tuniques de la paroi abdominale antéro-latérale. Dans notre cas, la tomodensitométrie abdominale avait posé le diagnostic en objectivant l'incarcération pariétale d'anse grêle à travers l'aponévrose de la ligne semi-lunaire. La hernie était en position sous et para ombilicale droite. Elle avait également permis d'apprécier la sévérité de l'occlusion en objectivant le défaut de rehaussement pariétal des anses contenues dans le sac herniaire. Nous avons ainsi retenu une occlusion compliquée d'une ischémie artérielle. La présence de ces signes de souffrance intestinale a été un facteur pronostique essentiel permettant de hâter la prise en charge thérapeutique.

Le traitement de la hernie de Spiegel est chirurgical [1]. Chez notre patiente l'exploration chirurgicale avait trouvait un sac herniaire, au collet étroit (3 cm), contenant de l'épiploon et une anse grêle nécrosée sur 5 cm. Les suites opératoires étaient simples.

## Conclusion

La hernie de spiegel est une affection rare, de diagnostic clinique parfois difficile. L'étranglement reste une complication rare mais grave pouvant mettre en jeu le pronostic vital. La tomodensitométrie abdominale permet de poser le diagnostic, de rechercher des complications notamment l'étranglement et son retentissement. Le traitement est chirurgical.
